# Atomic-level bonding inducing strong metal–support interaction

**DOI:** 10.1093/nsr/nwaf454

**Published:** 2025-10-22

**Authors:** Yan Du, Huanyu Jin, Hui-Ming Cheng

**Affiliations:** Institute of Technology for Carbon Neutrality, Shenzhen Institutes of Advanced Technology, Chinese Academy of Sciences, China; Institute of Technology for Carbon Neutrality, Shenzhen Institutes of Advanced Technology, Chinese Academy of Sciences, China; Institute of Technology for Carbon Neutrality, Shenzhen Institutes of Advanced Technology, Chinese Academy of Sciences, China

Strong metal–support interaction (SMSI) has recently emerged as a frontier in heterogeneous catalysis, which profoundly influences the adsorption behavior, interfacial architectures and electronic state of supported metals [1–3]. In particular, SMSI between single atoms and supports enables the uniform dispersion and robust stabilization of metal species, suppresses aggregation and carbon deposition, and maximizes the utilization of interfacial active centers, thereby delivering outstanding catalytic performance in various chemical reactions [4,5]. However, achieving a comprehensive understanding of SMSI remains challenging, given that the complex interplay between metal dispersion, interfacial bonding and the support structure is difficult to disentangle.

**Figure 1.**
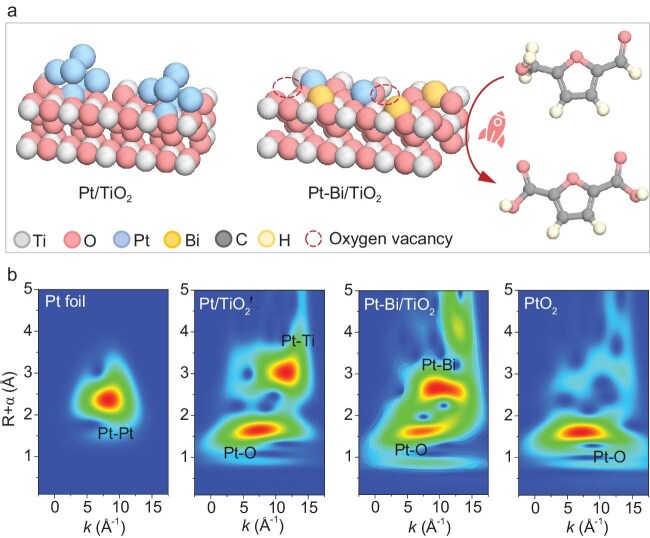
(a) Schematic diagram of Pt/TiO_2_ and Pt–Bi/TiO_2_. (b) Pt L3-edge XAS spectra: wavelet transform for the k3-weighted Pt L3-edge EXAFS. For comparison, the XAS spectra of standard Pt foil and PtO_2_ are also included. Reproduced with permission [6].

Recently, the research groups of Prof. Tianyi Ma from RMIT University, Prof. Yuanshuai Liu from Qingdao Institute of Bioenergy and Bioprocess Technology, Chinese Academy of Sciences, *et al*. collaboratively uncovered SMSI driven by Pt–O–Bi bonding, which dramatically improved the catalytic activity in the biomass-upgrading process [6]. This work synthesized Pt–Bi/TiO_2_ catalysts featuring a mesoporous anatase crystal structure via an *in situ* one-pot evaporation-induced self-assembly method. Structural analysis indicated that Bi incorporation effectively facilitates Pt species dispersion, resulting in a catalyst surface predominantly dominated by Pt single atoms (SAs) uniformly distributed on the Bi/TiO_2_ support (Fig. 1a).

Carbon monoxide diffuse reflectance infrared Fourier transform spectroscopy uncovered the existence of SMSI in the Pt–Bi/TiO_2_ system. A key finding was the complete disappearance of the CO adsorption signal on Pt SAs after high-temperature reduction, marking the onset of SMSI. This phenomenon ascribes electron transfer from the partially reduced TiO_2_ support to Pt SAs, generating negatively charged Pt^δ^^–^ species, coupled with a reversible encapsulation of Pt sites by support-derived layers. The subsequent restoration of the CO signal upon oxidation confirmed the dynamic and reversible nature of this encapsulation, which effectively prevents sintering of the metal atoms. In addition, the dramatic lowering

of the SMSI onset temperature for Pt–Bi/TiO_2_compared with Pt/TiO_2_ proved the greatly enhanced metal–support interaction. X-ray absorption spectroscopy (XAS) analysis at the Pt L3-edge provided direct spectroscopic evidence for the atomic-level bonding structure that induces the enhanced SMSI effect. The X-ray absorption near-edge structure spectra showed that Pt species maintain highly oxidized states. It is noteworthy that the absence of Pt–Pt bonds is shown by the extended X-ray absorption fine structure (EXAFS) spectra, verifying the dominant single-atom dispersion of Pt (Fig. 1b). The detection of a distinct Pt–Bi scattering path in the bimetallic catalyst directly demonstrated the formation of Pt–O–Bi interfacial bonding. Furthermore, quantitative fitting of the EXAFS data showed a lengthened Pt–O bond and a decreased coordination number in Pt–Bi/TiO_2_, indicating significant structural distortion and the presence of abundant oxygen vacancies facilitated by Bi incorporation. The SMSI induced by these structural features contributes to boosting the activation of molecular oxygen and reducing the energy barrier for the base-free oxidation of biomass-derived 5-hydroxymethylfurfural, thus significantly accelerating the reaction rate.

In summary, this paper provides in-depth mechanistic insight into SMSI regulation at the atomic scale and opens a promising avenue for the precise design of highly efficient catalysts for sustainable biomass valorization.
